# Overexpression of the Fibroblast Growth Factor Receptor 1 (FGFR1) in a Model of Spinal Cord Injury in Rats

**DOI:** 10.1371/journal.pone.0150541

**Published:** 2016-03-25

**Authors:** Barbara Haenzi, Katharina Gers-Barlag, Halima Akhoundzadeh, Thomas H. Hutson, Sean C. Menezes, Mary Bartlett Bunge, Lawrence D. F. Moon

**Affiliations:** 1 Neurorestoration Group, Wolfson Centre for Age-Related Diseases, King’s College London, London, SE1 1UL, United Kingdom; 2 Miami Project to Cure Paralysis, Departments of Cell Biology, Neurological Surgery and Neurology, University of Miami Miller School of Medicine, Miami, FL, 33136, United States of America; Inserm, FRANCE

## Abstract

Spinal cord injury (SCI) is a severe condition that affects many people and results in high health care costs. Therefore, it is essential to find new targets for treatment. The fibroblast growth factor receptor 1 (FGFR1) signalling pathway has a history of being explored for SCI treatment. Several groups have examined the effect of high availability of different FGFR1 ligands at the injury site and reported corticospinal tract (CST) regeneration as well as improved motor functions. In this study, we investigated overexpression of the FGFR1 in rat corticospinal neurons *in vivo* after injury (unilateral pyramidotomy) and in cerebellar granule neurons (CGNs) *in vitro*. We show that overexpression of FGFR1 using AAV1 intracortical injections did not increase sprouting of the treated corticospinal tract and did not improve dexterity or walking in a rat model of SCI. Furthermore, we show that overexpression of FGFR1 *in vitro* resulted in decreased neurite outgrowth compared to control. Thus, our results suggest that the FGFR1 is not a suitable therapeutic target after SCI.

## Introduction

Spinal cord injury (SCI) is a condition that affects 250,000 to 500,000 people worldwide (World Health Organisation 2013). Axons of the central nervous system (CNS) have a very low level of spontaneous regeneration compared to axons of the peripheral nervous system. Regeneration of the CNS is inhibited by extrinsic factors (e.g., inhibitory proteoglycan and myelin-associated factors), as well as intrinsic factors (e.g., lack of regeneration-associated gene expression). Since there is a shortage of therapies to increase regeneration after SCI, research aimed at identifying new targets for therapy is very important. The FGFR pathway has a history of being investigated as a therapeutic target. Various studies have demonstrated beneficial effects of delivering FGFR1 ligands to the injury site following SCI [1–9]. Peripheral nerve grafts and slow releasing matrices filled with FGF1 have been supplied to the injury site either alone [1] or in combination with agents to alter the inhibitory environment of the scar tissue [2, 8]. These approaches have led to improved CST regeneration and motor function. Furthermore, FGF2 has been demonstrated to improve CST growth when delivered close to the injury site after SCI [3, 4, 9]. Similar improvement of CST growth has been shown by overexpression of the FGFR1 ligand L1 that leads to activation of the endocannabinoid system [5–7].

The fibroblast growth factor receptor 1 (FGFR1) is one of four different FGF receptors, named FGFR1-4, FGF1-3 exist in two different splice variants [10]. So far 22 fibroblast growth factor (FGF) ligands have been identified. FGF1 and FGF2 are both secreted ligands, signal in a para- or autocrine fashion and bind all four receptors [10]. They are abundant in the intact and injured nervous system [11, 12] and many therapeutic approaches concentrate on these two ligands. In addition to the FGF ligands, there are a number of adhesion molecules, such as Ncam, N-cadherin, and L1, that have been shown to activate the FGFR pathway in the nervous system [13–15]. Activation of the FGFR pathway via adhesion molecules has been found to result in phospholipase Cγ (PLCγ) activation [16] leading to activation of the endocannabinoid system [17]. This in turn has been shown to stimulate neurite outgrowth *in vitro* [18, 19]. Here, we investigated whether overexpression of the common receptor of these ligands, FGFR1, in corticospinal neurons increases sprouting of the treated neurons and improves dexterity or walking in a rat model of SCI. Furthermore, we shed light on the underlying mechanism by which FGFR1 signalling affects neurite outgrowth *in vitro* in cerebellar granule neurons (CGNs).

## Results

**Fig 1:**
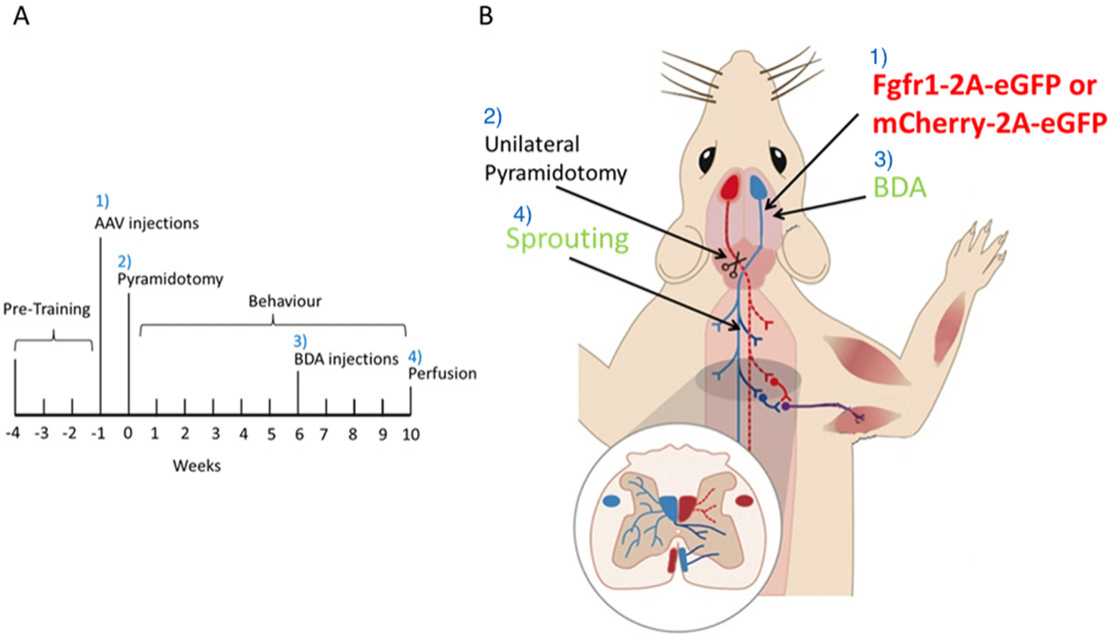
Timeline and scheme of in vivo experiment. A) Rats were pre-trained on the Montoya staircase test and the horizontal ladder for three weeks. One week before the SCI (pyramidotomy) animals were injected with AAVs overexpressing FGFR1 or mCherry into the sensorimotor cortex innervating the less preferred paw. During spinal cord surgeries the medullary pyramids were cut unilaterally in the brainstem innervating the preferred paw of all animals. Three days post-surgery the animals were assessed behaviourally and thereafter every week for ten weeks. Four weeks prior to the end of the study all animals were injected with BDA on the same side as to the vector injection. B) Scheme of the different surgical procedures. 1) Injection of AAVs expressing either CMV-FGFR1-2A-EGFP or CMV-mCherry-2A-EGFP, 2) unilateral pyramidotomy, 3) BDA injection for axon tracing, and 4) counting fibres that sprouted over the cervical midline.

**Fig 2:**
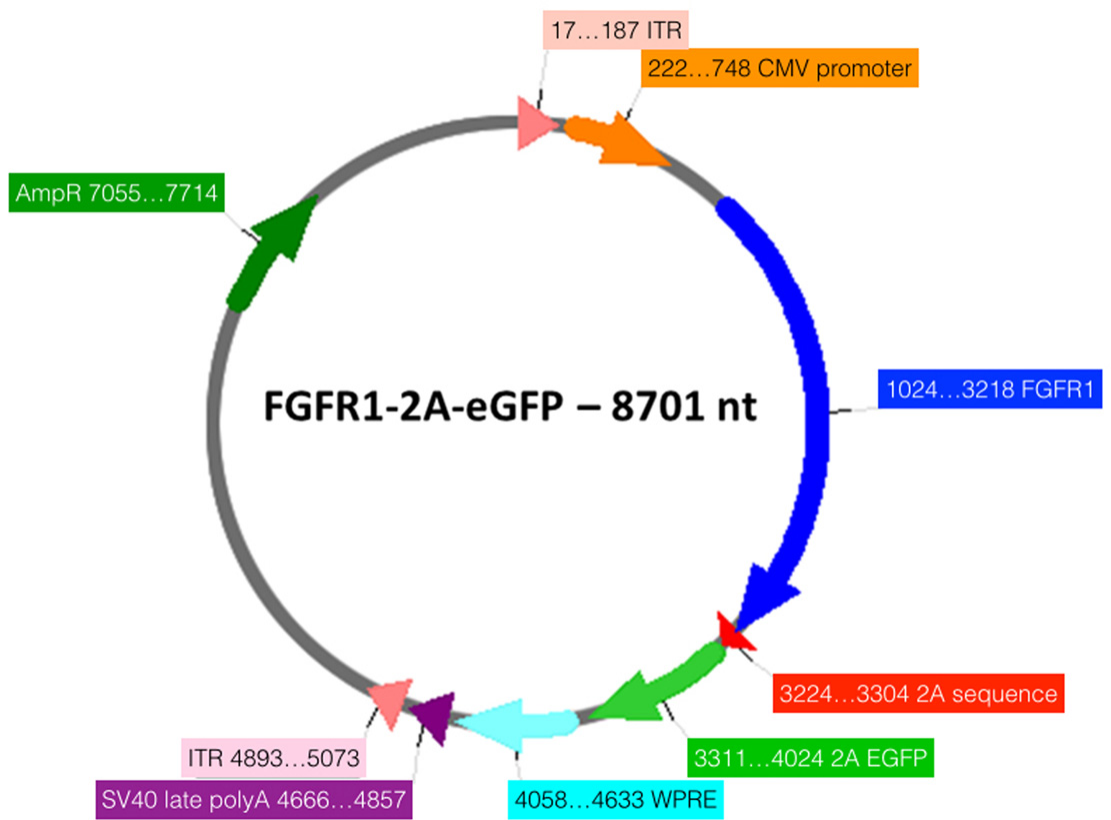
Schematic diagram of the CMV- FGFR1-2A-eGFP vector. The two genes are separated by a 2A sequence to achieve expression of two separate proteins from the single cytomegalovirus (CMV) promoter (19). ITR: Inverted Terminal Repeat Sequences; CMV: cytomegalovirus; WPRE: woodchuck hepatitis virus post-transcriptional regulatory element; AmpR: ampicillin resistance. Created using Serial Cloner 2.6.

We investigated overexpression of the FGFR1 in an *in vivo* model of SCI. The time-line of this experiment is depicted in Fig 1A. Rats were randomised to treatment, and all behavioural experiments were performed in a blinded manner. Animals were pre-trained for three weeks on the Montoya staircase test and on a horizontal ladder with irregularly spaced rungs. During this period the preferred forepaw was identified according to the staircase test performance. We produced an adeno-associated viral vector (AAV) serotype 1 overexpressing FGFR1 and EGFP or mCherry and EGFP from the following bicistronic vectors: CMV-FGFR1-2A-EGFP (Fig 2) CMV-mCherry-2A-EGFP. The two genes are separated by a 2A sequence to achieve expression of two separate proteins from the single CMV promoter [20]. AAV-CMV-FGFR1-2A-EGFP or AAV-CMV-mCherry-2A-EGFP was injected into the motor cortex controlling the less preferred forepaw. One week after AAV injection all rats underwent unilateral injury of the corticospinal tract (CST) (unilateral pyramidotomy) controlling the preferred forepaw (Fig 1B). We used AAV serotype 1 because we had perviously shown that this transduces cortical neurons including corticospinal neurons [21]. We injected it intracortically to test the hypothesis that unlesioned corticospinal axons would sprout into the affected hemicord after pyramidotomy.

**Fig 3:**
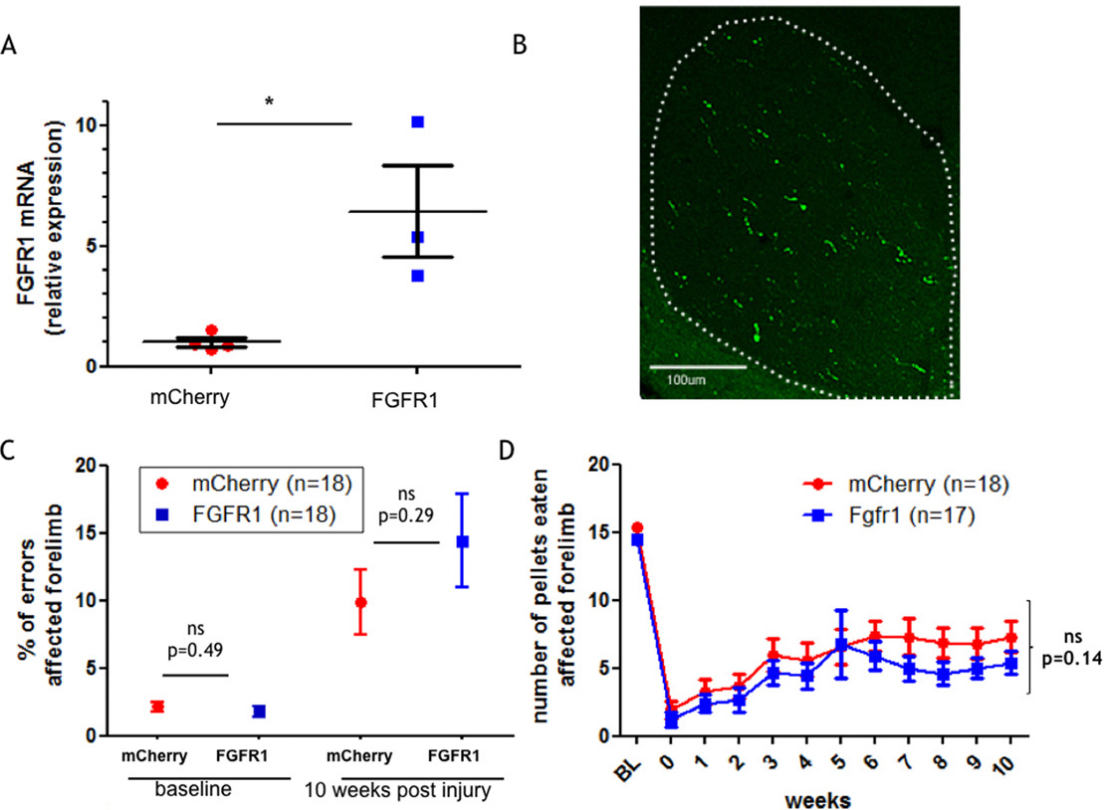
FGFR1 overexpression did not increase dexterity in the Montoya Staircase or reduce errors on the horizontal ladder. A) Cortical expression of FGFR1 transcript in mCherry and FGFR1 injected animals. A separate cohort of rats was used for this experiment. qRTPCR was performed with RNA extracted 1 to 2.5 weeks after injection of AAV. The expression values of FGFR1 expression was normalised to GAPDH expression. Graph shows means ± SEM, p = 0.02. B) A representative picture of a coronal section of the spinal cord at the level of C2 stained using antibodies against GFP, 10 weeks after pyramidotomy. GFP positive fibres were found unilaterally in the dorsal columns, confirming expression in CST axons. Dotted line shows outline of dorsal columns on the side. Scale bar, 200μm. C) All animals were tested on the horizontal ladder before surgery (baseline) and after 10 weeks. Depicted is the percentage of errors made when crossing the ladder. Graph shows means ± SEM. D) All animals were tested on the Montoya staircase test three days after surgery and thereafter weekly for ten weeks. BL = base line. Graph shows means ± SEM.

We confirmed the functionality of the injected AAV by validating overexpression of *FGFR1* in animals that were injected with the AAV-CMV-FGFR1-2A-EGFP relative to control animals (AAV-CMV-mCherry-2A-EGFP) by quantitative reverse transcription PCR since antibodies against FGFR1 are not suitable for immunostaining. Our results show that in motor cortices that were isolated from animals injected with AAV-CMV-FGFR1-2A-EGFP the expression level of *FGFR1* is about six fold higher compared to AAV-CMV-mCherry-2A-EGFP injected animals (p = 0.02) (Fig 3A). To confirm that the CMV-FGFR1-2A-EGFP construct is expressed in the CST we stained coronal sections of the C2 spinal cord. Due to the lack of trustworthy antibodies against FGFR1 we stained using antibodies against GFP. Corticospinal axons expressed the reporter transgene (Fig 3B). This is consistent with previous work from our group [22, 23]. This will underestimate the number of FGFR1 overexpressing CST fibres since our group has shown that the second gene in these bicistronic vectors can be expressed less than the first gene and that the reporters are not transported into the axon collaterals within grey matter [22, 23] (Fig 3B). We then investigated a potential functional effect of FGFR1 overexpression after SCI. All rats were tested on a horizontal ladder with irregularly spaced rungs. Rats had to cross the ladder three times and the performance was averaged over the 3 runs. Fig 3C depicts the percentage of errors made by the affected forelimb at baseline and then 10 weeks post-injury. The baseline level for the percentage of errors made by crossing the ladder was around 2% for control and FGFR1 overexpressing animals. The number of errors made by the affected forelimb increased up to 10–15% 10 weeks post-injury. However, we did not find a significant difference between control and FGFR1 overexpressing animals. We also tested all rats on the Montoya staircase test to investigate dexterity. Rats were offered three sugar pellets on each step on both sides. The number of sugar pellets consumed was recorded on each side of the staircase for each rat. We show here the data for the affected forelimb. All rats showed a considerable deficit after SCI and partial spontaneous recovery over the tested period. The staircase test revealed a tendency of AAV-CMV-FGFR1-2A-EGFP injected animals to retrieve fewer pellets compared to control animals. However this difference did not reach significance (Fig 3D).

**Fig 4:**
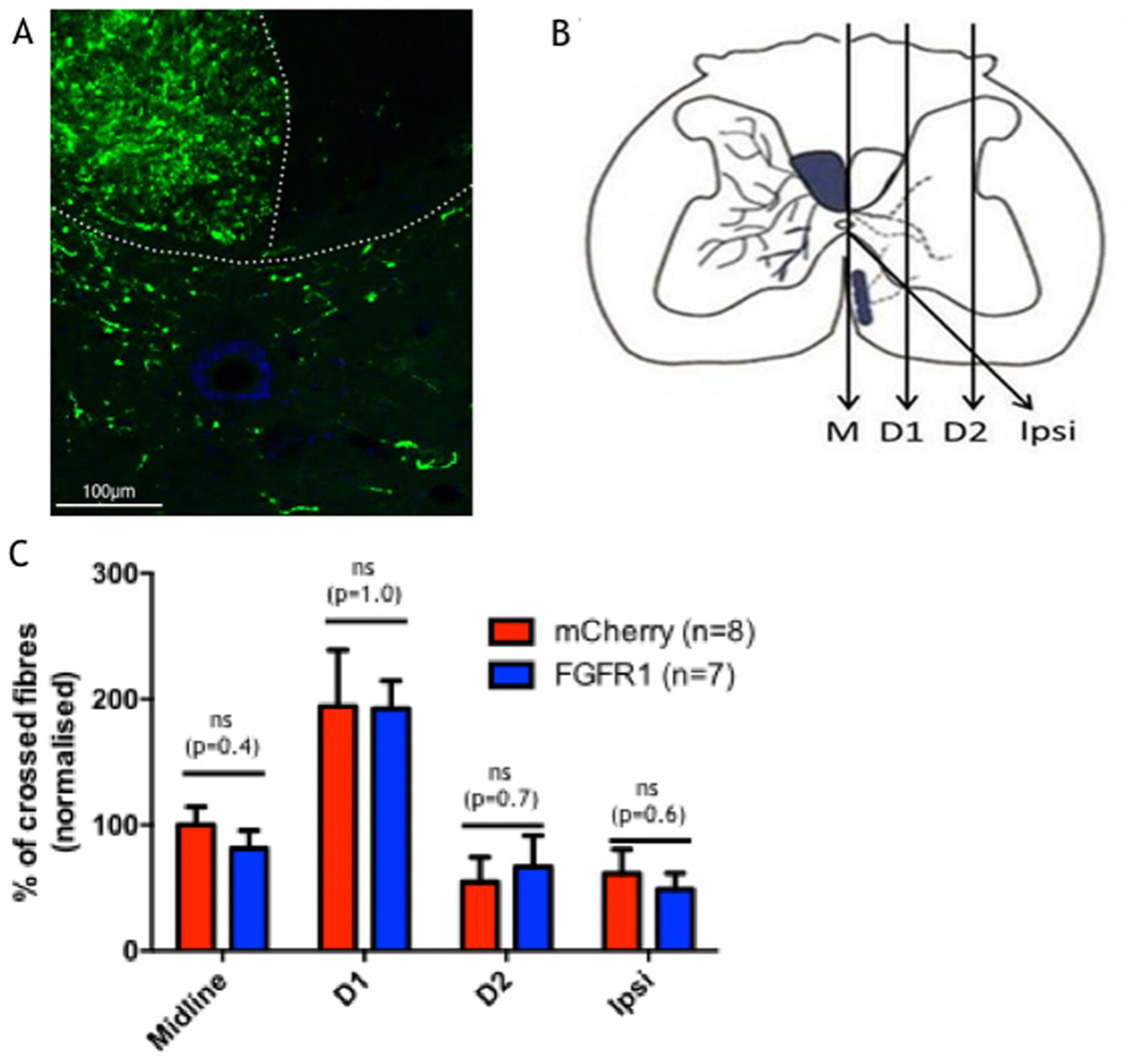
Tracing of intact corticospinal axons. A) A representative picture of BDA traced C7 spinal cord Dotted line shows outline of the grey matter and dorsal column. Scale bar, 100μm. B) Crossing of intact corticospinal fibres was assessed by counting injecting BDA in the motor cortex innervating the unlesioned forepaw. Fibres that crossed over were counted at C7 at the midline, two different distances from the midline (D1 and D2). Sprouting of the non-decussated fibres was counted on the line called “Ipsi”. C) Number of fibres that crossed a given line in C7 for control and FGFR1 overexpressing animals.

We also examined if overexpression of FGFR1 resulted in increased sprouting of the intact CST over the cervical spinal midline. Previous experiments have shown that the EGFP expression from the bicistronic AAV used is not strong enough to allow tracing of the whole axon [22], therefore, we injected all rats 4 weeks prior to perfusion with the anterograde tracer Biotinylated Dextran Amine (BDA) (refer to the time line of the experiment in Fig 1A). The injections were performed into the motor cortex that normally controls the less preferred paw. The C7 spinal cord segment was processed for staining and the BDA positive fibres were visualised by immunolabelling (Fig 4A). Crossing over of intact CST fibres into the affected spinal hemicord was assessed at three different planes and the “ipsi” boundary where non-decussated CST fibres enter the grey matter from the ventral white matter (Fig 4B). We investigated if the fibres crossed the midline (M), and if they did so, how far they grew (at two different distances from the midline, D1 and D2). Analysis of the number of sprouted fibres showed that there was no difference between the two groups (Fig 4C). These results are in line with our observations that there is no functional difference between the two groups. In summary, we conclude that overexpression of FGFR1 following SCI did not improve dexterity or walking, or increase sprouting of the treated corticospinal tract in a rat model of SCI.

**Fig 5:**
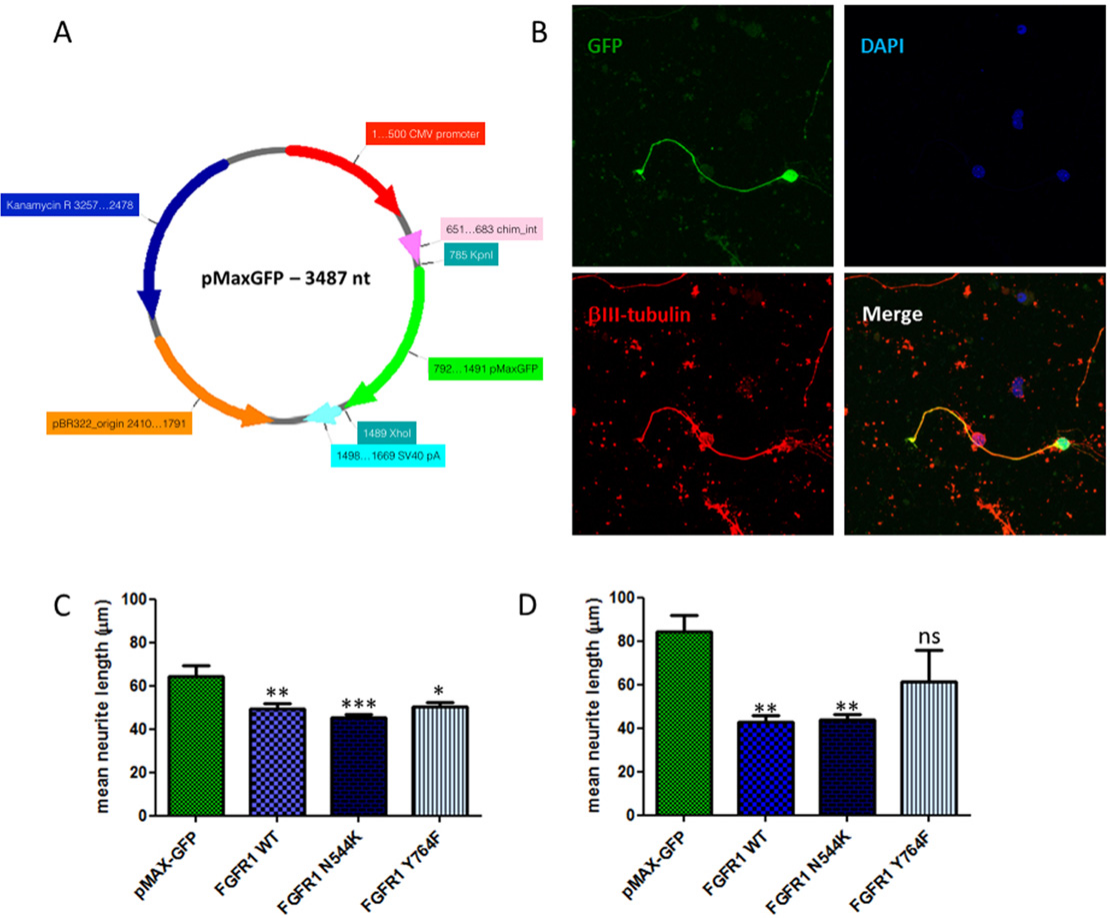
Wild type and mutant FGFR1 reduced neurite length on growth permissive PLL and growth inhibitory CSPGs when compared to control cells. A) Schematic diagram of the pMaxGFP vector highlighting distinct features. CMV: cytomegalovirus. B) Representative pictures of cerebellar granule neurons grown on CSPG. C,D) Cells were transfected with 1μg pMaxGFP and 4μg of either wild type or mutant FGFR1 by electroporation and then cultured on PLL (C) or CSPGs (D) for 48 hours. Control cells were transfected with 1μg pMaxGFP. Cells were stained for βIII tubulin and DAPI and analysed for neurite outgrowth by fluorescence microscopy. Data are mean ± SEM (n = 8) and significance is shown for Dunnett’s post hoc test values when groups were compared to cells transfected with pMaxGFP alone. *P < 0.05; **P < 0.01; ***P < 0.001; ns–not significant.

To investigate the underlying mechanism of the FGFR1 pathway on neurite outgrowth we examined FGFR1 overexpression *in vitro*. We isolated CGNs from rat pups at post-natal day 7 to 9 to investigate neurite outgrowth. A single cell suspension of CGNs was electroporated with a bicistronic plasmid overexpressing FGFR1 and EGFP (CMV-FGFR1-2A-EGFP) (Fig 2). We and others find that of those neurons which survive electroporation, only a proportion express the transgene after electroporation of plasmids [24–27]. Because we need to measure neurite growth only in those neurons which express the FGFR1 transgene, we co-electroporate neurons with a green fluorescent reporter (pMaxGFP), which enables us to detect transgenic neurons, as described previously [26, 28]. To ensure a high level of expression of green fluorescence, cells were co-electroporated with pMaxGFP (Fig 5A) because prior work from our group showed that the 2A-EGFP sequence did not adequately label all the neurites. Control cells were electroporated with pMaxGFP only. After electroporation cells were grown on a growth permissive poly-L-lysine (PLL) substrate or on a growth inhibitory chondroitin sulphate proteoglycan (CSPG) substrate for 48 hours. These experiments revealed that overexpression of wild-type (WT) FGFR1 reduces neurite outgrowth by 20% when cells were grown on PLL (p = 0.01) (Fig 5C), and by 50% when grown on CSPGs (p = 0.003) (Fig 5D). We also overexpressed a mutated form of FGFR1, which harbours a lysine instead of an asparagine at position 544 (N544K). This mutant has been shown to have increased kinase activity [29, 30]. Our results show that overexpression of FGFR1-N544K results in the same degree of downregulation of neurite outgrowth as with overexpression of the WT FGFR1 (p>0.05) (Fig 5C & 5D). To further understand the mechanism by which FGFR1 reduces neurite outgrowth we mutated tyrosine 764 to phenylalanine (Y764F) in order to prevent phosphorylation of FGFR1 at this position, and therefore binding and activation of PLCγ [31, 32]. Our data show that overexpression of the FGFR1-Y764F mutant did not significantly alter neurite outgrowth compared to WT FGFR1 overexpression (p>0.05) (Fig 5C & 5D).

## Discussion

We show here that overexpression of FGFR1 *in vitro* in CGNs reduces neurite outgrowth compared to control cells. The underlying mechanism for outgrowth inhibition upon FGFR1 overexpression needs further investigation. We have shown that inhibition of PLCγ signalling downstream of overexpressed FGFR1 does not rescue the observed reduction in neurite outgrowth.

For our *in vitro* experiments we grew the transfected CGNs for 48 hours on growth permissive PLL or on growth inhibitory CSPG coated 96-well plates. Neurites were longer on CSPG (80μm) compared to PLL (60μm) coated plates. This was unexpected as we and others have previously shown that CGN neurite lengths are shorter on CSPG, however, the finding was very robust across independent experiments and we believe that it might have been due to the particular batches of the CSPG that we used.

We have also shown that overexpression of FGFR1 in the motor cortex of rats with a contralateral lesion of the corticospinal tract in the pyramids is not beneficial for sprouting of intact fibres over the midline and does not lead to increased functional regeneration as measured by the Montoya staircase and horizontal ladder test.

A range of other studies have shown that FGFR1 ligand supplementation at the site of SCI increases neurite outgrowth and is functionally beneficial [1–8]. Therefore, we hoped to combine the beneficial effect of various FGFR1 ligands by overexpression of the receptor. Interestingly, overexpression of FGFR1 did not lead to a functional benefit and/or neurite outgrowth. Possible explanations for the lack of neurite outgrowth might be that the previously studied ligands do not only bind FGFR1, but also other FGF receptors, such as FGFR2. The activation of other receptors in addition to FGFR1 may be essential in order to result in a beneficial outcome after injury. Indeed, during preparation of this manuscript, another group showed that FGFR1 and FGFR2 are constitutively expressed in the motor cortex of mice and are required for the limited degree of spontaneous recovery after SCI that occurs [33]. Our data extend this work by showing that overexpression of FGFR1 by itself does not further enhance spontaneous recovery after SCI. In addition, we report a worse outcome for neurite outgrowth following FGFR1 overexpression *in vitro*. This might be due to the overexpressed FGFR1 sequestering adaptor proteins away from other pro-neurite outgrowth pathways and therefore inhibiting these pathways. Indeed, it has been shown by others that overexpression of FGFR1 inhibits nerve growth factor signalling via TRKA, the receptor for the nerve growth factor (NGF). TRKA signalling is dependent on the FGF receptor substrate 2 (FRS2) [34]. PLCγ activation downstream of FGFR1 signalling has been described to induce neurite outgrowth [35, 36]. We therefore overexpressed a mutant of FGFR1 (Y764F) that is unable to activate PLCγ. However, this mutant had no effect on neurite outgrowth compared to WT FGFR1 overexpression. This suggests that the reduced neurite outgrowth that we observed upon FGFR1 overexpression is not mediated via PLCγ signalling.

In conclusion, we found that overexpression of FGFR1 in the motor cortex of rats after SCI is not beneficial in a rat model of spinal cord injury. This might be due to depletion of adaptor proteins from other pro-regenerative signalling pathways. Further studies are needed to elucidate the exact mechanism. We believe that overexpression of a combination of pro-regenerative receptors mutated for enhanced function could lead to positive effects.

## Material and Methods

All procedures were in accordance with the UK Home Office guidelines and Animals (Scientific Procedures) Act of 1986 and have been approved by the AWERB and Home Office of the UK. Anaesthesia was performed using Isoflurane, Euthanasia was performed using an overdose with Euthatal

*Cerebellar granule neuron culture and electroporation*: Postnatal day 7–9 (P7–9) CGNs were prepared as previously described [26]. We originally identified FGFR1 as a candidate pro-regenerative gene using CGNs in a high content screening study of more than 500 plasmids (unpublished). In that previous work, we used CGNs because one may obtain large numbers of these neurons easily for screening hundreds of different candidate genes. In the present work we decided to continue using CGNs for continuity (rather than to change to a new cell type such as cortical neurons). Briefly, the cerebellae of P7-9 rat pups were isolated, the meninges removed and the cerebellae finely diced with a razor blade before being incubated with 5 ml 0.05% trypsin/EDTA in calcium and magnesium free medium (CMF) containing 0.4 mg/ml KCl, 0.06 mg/ml KH2PO4, 7.65 mg/ml NaCl, 0.35 mg/ml NaHCO3, 0.048 mg/ml Na2HPO4, 2.38 mg/ml HEPES in sterile water (pH 7.2) for 15 min at 37°C. The trypsin/EDTA was deactivated using an equal volume of 10% fetal bovine serum (FBS) in CMF. The cell pellet was mechanically triturated in the presence of 0.5 ml 5 mg/ml DNase I (Sigma) in 2 ml CMF. The cells were left to settle for 5 min before 1.5 ml of supernatant was harvested and the cells collected by centrifugation at 100×g for 5 min. The cell pellet was resuspended in 5 ml serum free media containing Neurobasal media (Invitrogen) supplemented with, 2% B27 (Invitrogen), 25 mM KCl (Sigma), 100 U/ml penicillin and 100 μg/ml streptomycin (Invitrogen), 3 mg/ml D-glucose (Sigma), 2 mM L-glutamine (Sigma). Cells were counted using a hemocytometer and resuspended in internal neuronal buffer containing 135 mM KCl, 2 mM MgCl2, 10 mM HEPES, 0.2 mM CaCl2, 5 mM ethylene glycol tetraacetic acid (EGTA) and sterile water (pH 7.3). The cells were mixed with either 1μg pMaxGFP DNA for control cells or 1 μg pMaxGFP and 4μg CMV-FGFR1-2A-EGFP DNA on a 96 well plate. Electroporation was performed with a HT-200 plate handler connected to an ECM 830 square-wave pulse generator (BTX Harvard Apparatus). The pulse generator was connected to a TDS 1002 oscilloscope (Tektronix, USA) to monitor the delivery of the required pulses. Parameters used were 300 V and a 1 ms pulse. Directly after electroporation Hibernate E (Thermo Fisher Scientific A12476-01) was added to the cells before they were plated on PPL or CSPG coated dishes.

*Measurement of neurite outgrowth*: 48 hours after electroporation and growth on PLL or CSPG, cells were fixed with cold 4% paraformaldehyde (PFA) in PBS. Cells were stained overnight at room temperature with an anti βIII tubulin antibody (Promega, USA) diluted in PBS containing 10% normal goat serum (Invitrogen) and 0.2% Triton-X-100. The next day, cells were incubated for 1 hour in goat anti mouse IgG conjugated to Alexa Fluor 546 (Invitrogen, USA) and 4’, 6-diamidino-2-phenylindole (DAPI), diluted in PBS containing 0.2% Triton-X-100. Images were taken with an IN Cell Analyser 1000 with a 10x camera objective and 10x Nikon ApoPlan objective (GE Healthcare Life Sciences, UK). Calculation of neurite length was achieved using the IN Cell Developer Toolbox software. Transfected neurons were identified by GFP expression.

*DNA preparation*: The Mus Musculus *FGFR1* (BC033447.1) cDNA clone was purchased pre-cloned into the pCMVSPORT6 (Source Bioscience, Nottingham, UK) which uses the RNA polymerase II human CMV promoter to drive transgene expression. The pMaxGFP plasmid was purchased from Amaxa, which uses the CMV promoter to drive expression of maxGFP (Amaxa, UK). The pCMVSPORT6-mCherry plasmid was a kind gift (Dr. Willie Buchser, Prof. Vance Lemmon and Prof. John Bixby, University of Miami Miller School of Medicine). mCherry was cloned out of this vector and transferred into the psubCMV-2A-WPRE vector as described below.

*Bicistronic plasmid construction*: The psubCMV-2A-WPRE AAV transfer plasmid was a kind gift from Dr. Hansruedi Bueler, University of Kentucky. The coding sequences for mouse FGFR1 (BC033447.1), mCherry and EGFP were amplified using PCR and cloned in frame either upstream (FGFR1 or mCherry) or downstream (EGFP) of the Foot and mouth disease virus (FMDV) 2A sequence as described before [22]. The PCR amplification primers were designed so that the upstream coding sequence lacked a translation stop codon while the downstream coding sequence lacked a translation initiation codon. Both plasmids contained a CMV promoter to drive expression and a woodchuck hepatitis virus post-transcriptional regulatory element (WPRE). The two plasmids that were generated (psubCMV-FGFR1-2A-Egfr-WPRE and psubCMV-mCherry-2A-EGFP-WPRE) were sequenced to confirm successful cloning.

*Site-directed mutagenesis*: Site-directed mutagenesis was performed according to the QuikChange Lightning Site-Directed Mutagenesis Kit (Agilent Technologies, Wokingham, UK). The mutations were introduced into the psub-CMV-FGFR1-2A-EGFP vector by performing PCR using the following mutagenic primers (the triplets coding for the desired mutated amino acid are underlined and the desired mutated nucleotides are highlighted in bold): N544K-forward 5’ GCA CAA GAA TAT CAT CAA **G**CT TCT GGG AGC GTG C 3’ N544K-reverse 5’ GCA CGC TCC CAG AAG **C**TT GAT GAT ATT CTT GTG C 3’; Y764F-forward 5’ GCA CAA GAA TAT CAT CAA **G**CT TCT GGG AGC GTG C 3’ Y764F-reverse 5’ ACA GGT CCA GA**A** ACT CCT GGT TGG AGG TCA AGG 3’. PCR reactions contained 5μl 10X reaction buffer, 50ng dsDNA template, 125ng each forward and reverse mutagenic primer, 1μl dNTP mix, 1.5μl QuikSolution reagent, ddH_2_0 and 1μl QuikChange Lightning enzyme in a final volume of 50μl. The PCR cycle conditions were as follows: 95°C for 2 minutes; 95°C for 20 seconds, 60°C for 10 seconds, 68°C for 5 minutes, 18 cycles, 68°C for 5 minutes. Sequencing confirmed successful clones.

*Quantitative reverse transcription PCR (qRTPCR)*: We used tissues from rats that were euthanised between 1 and 2.5 weeks after AAV injection. Injected rat cortex was snap frozen after collection and stored at -80°C. Cortices were homogenised using a GentleMACS program RNA_02.1 (Miltenyi Biotech). RNA was extracted using Trizol (Invitrogen). Reverse transcription was performed from1ng RNA using Superscript III Reverse transcriptase (Invitrogen) and random primers (Invitrogen) as per the manufacturer’s instruction. FGFR1 expression was assessed using quantitative PCR (qPCR). qPCR was performed with a LightCycler 480 II from Roche and with the LightCycler 480 SYBR Green I Master (04887352001, Roche). Values of expression were calculated using the values for slope and intercept as calculated from the standard curve on the same plate. The PCR program used was as follows: Pre-incubation: 95°C for 5 minutes, ramp rate 4.8C/s. Amplification (45 cycles): 95°C for 10 seconds, ramp rate 4.8 C/s, 60°C for 10 seconds, ramp rate 2.5°C/s, 72°C for 10 seconds, ramp rate 4.8°C/s. All quantitations were normalized to rat Gapdh. Primers: mouse *FGFR1* 5'-GGTTGACCGTTCTGGAAGC-3', 5'-GCCCCGGTGCAGTAGATA-3'. Rat *Gapdh* 5'-GTTACCAGGGCTGCCTTCTC-3', 5’-ACCAGCTTCCCATTCTCAGC-3’

*Experimental in vivo design*: The experimental design is presented in Fig 1. All procedures, behavioural testing and analysis were performed using a randomised block design and in a blinded fashion: codes were only broken after the end of the study.

*Adeno-associated viral vector injection*: Adeno-associated viral vectors (AAVs) serotype 1 expressing either *mCherry* or *FGFR1* were titre matched at 2.44E+12 GC/ml (Genomic Copies/ml) and injected into the sensorimotor cortex that controls the non-preferred forepaw. Seven holes were drilled into the skull using a dental drill at the following anterioposterior (AP) and mediolateral (ML) coordinates relative to Bregma: 1) AP: +1.0 mm, ML: 1.5 mm; 2) AP: +0.5 mm, ML: 2.5 mm; 3) AP: +1.5 mm, ML: 2.5 mm; 4) AP: +0.5 mm, ML: 3.5 mm; 5) AP: +2.0 mm, ML: 3.5 mm; 6) AP: −0.5 mm, ML: 3.5 mm; 7) AP: +3.5mm, ML: 2.0 mm. The vector was injected using a Hamilton syringe and a 34G needle (point style 2; Hamilton). The needle was slowly lowered 1.5 mm below the cortical surface and 0.5 μl of vector was injected at a rate of 0.25 μl per 10 seconds with a pause of 1 min after each infusion. The scalp was then sutured and analgesic given as described below.

*Spinal cord injury surgery*: The preferred forepaw was identified in the Montoya staircase test. One week after AAV injection the CST innervating the preferred forepaw was cut unilaterally before it decussates at the level of the pyramids in the brainstem as described earlier [37]. Briefly, animals were anesthetised with isoflurane. 4% isoflurane in O_2_ was used for induction; 1.5% to 2% in O_2_ was delivered *via* a face mask to maintain anaesthesia. Rectal temperature was maintained at ~37°C using a homeothermic system. The analgesic Carprofen (5mg/kg) was administered subcutaneously. A 2–3 cm midline incision caudal to the chin was performed, and the muscular layer was blunt dissected. The trachea was slightly displaced to one side and muscles were further dissected until the skull became visible. In a modification of the surgery described previously [37], we did not cauterise any blood vessels and no retractors were used. Instead, the surgical field of interest was kept clear from surrounding tissue with forceps. The skull above the medullary pyramid corresponding to the preferred forepaw was opened up using a Friedman-Pearson rongeur (0.5mm cup, curved, 16221–14). The dura was cut and a 1.5mm wide and 1mm deep cut from the midline was performed in the pyramid as described in Kathe et al. [37]. After the cut, all bleeding was stilled and the animal was sutured. 5ml 0.9% saline was administered to each flank of the animal. Dopram was only used in cases where the animals had acute breathing problems during the procedure. Carprofen was given on the two consecutive days.

*Behaviour*: Rats were trained (for 3 weeks) and evaluated (10 weeks) on behavioural tasks. Preoperative baseline scores for the horizontal ladder were collected one and two weeks before surgery. Postoperative behaviour was performed on day 3 post-surgery and then weekly thereafter.

Horizontal ladder: The apparatus consisted of Plexiglas side walls, 1.2 m long, 50 cm high and width adjusted to approximately 2 cm wider than the animal to try and prevent turning. Metal rungs were placed at a height of 20 cm; they were spaced unequally (between 1 cm and 4 cm apart) and changed weekly to avoid improvement through pattern learning. Rats were videotaped crossing a 1m-length of the horizontal ladder weekly, 3 times per session. Any slight paw slips, deep paw slips and complete misses were scored as errors. The mean number of errors per step was calculated for each limb for each week.

Montoya staircase test: The test was performed as described earlier [38]. Rats were offered three sugar pellets on each step on both sides. Each rat was assessed during 10 minutes. The number of sugar pellets consumed was recorded on each side of the staircase for each rat. Consumed pellets are defined as total pellets offered on all 7 staircases minus displaced-but-not-eaten pellets and any remaining pellets. During pretraining, rats were food restricted 24 hours prior to testing. Once the rats were used to the sucrose pellets food restriction was stopped.

*Corticospinal tract tracing*: Anterograde tracer (Biotinylated Dextran Amine, BDA, 10,000 kDa, 10% in PBS, Invitrogen) was injected into the contralesional sensorimotor cortex six weeks after injury. Seven holes were drilled into the skull using a dental drill at the following anterioposterior (AP) and mediolateral (ML) coordinates relative to Bregma: 1) AP: +1.0 mm, ML: 1.5 mm; 2) AP: +0.5 mm, ML: 2.5 mm; 3) AP: +1.5 mm, ML: 2.5 mm; 4) AP: +0.5 mm, ML: 3.5 mm; 5) AP: +2.0 mm, ML: 3.5 mm; 6) AP: −0.5 mm, ML: 3.5 mm; 7) AP: +3.5 mm, ML: 2.0 mm. 0.5μl of 10’000 MW BDA per injection site was infused using a Hamilton syringe with a 34G needle (Hamilton part number 207434, 11 mm/point style 2). The needle was slowly lowered 1.5 mm below the cortical surface and BDA injected at a rate of 0.25 μl per 10 seconds with a pause of 1 min after each infusion. The scalp was then sutured and analgesic given as described above. Ten weeks post CST surgery (i.e., four weeks after BDA labelling), rats were terminally anesthetised with Sodium Pentobarbital (Euthatal) and perfused transcardially with PBS (NaCl, 137 mM; KCl, 2.7 mM; Na_2_HPO_4_, 4.3 mM; KH_2_PO_4_, 1.4 mM) for 2 minutes, followed by 500 ml of 4% paraformaldehyde (PFA) in PBS for 12 minutes. The spinal cord and brainstem were carefully dissected and stored in 4% paraformaldehyde in PBS for 24 hours and then transferred to 30% sucrose in PBS and stored at 4°C. The C7 spinal cord segment was embedded in optimal cutting temperature compound (OCT) and 40 μm transverse slices were cut using a freezing stage microtome (Kryomat, Leitz, Germany) and transferred into TBS/azide (100mM Tris, 15 mM NaCl, 0.5mM NaN_3_, pH 7.4) and stored at 4°C. Ten series of sections were cut and placed in 10 wells.

*Histology*: *For BDA staining*, *f*ree floating sections were incubated in 0.3% H_2_O_2_ in H_2_O (30 min). Sections were incubated in ABC vector (VectorLabs, UK) (30 min) then amplified using biotinyl tyramide (1:75, PerkinElmer, USA), then left overnight at room temperature on a shaker with extra avidin FITC (1:500, Sigma). Sections were washed between all steps. Sections were cover slipped with Mowiol.

Corticospinal axons were counted that were anterogradely traced with BDA and crossed the midline, D1, D2, or the “ipsi” boundary at C7. For each rat, the number of corticospinal axons per cord segment were calculated by counting the number of corticospinal axons in all sections in a series, normalising to the number of fibres labelled in the dorsal column and then multiplying by the total number of sections in the whole C7 segment and then divided by the number of sections counted [39].

The presented representative picture was taken using a Leica SPE scanning laser confocal microscope controlled with Leica LAS AF software.

For GFP positive CST fibres in the dorsal columns, free floating sections were blocked in 10% goat serum, incubated with an anti-GFP antibody (abcam, ab13970) over night. Next day sections were washed incubated with secondary goat anti chicken 488 (A-11039), washed and mounted in VECTASHIELD Antifade Mounting Medium with DAPI (Vector Laboratories, H-1200). Immunofluorescence was visualised using a Leica SPE scanning laser confocal microscope controlled with Leica LAS AF software.

*Statistics*: Results are expressed as mean ± Standard Error of Mean (SEM). For Figs 3A, 3B and 4C Student’s unpaired t-test was used to determine significant values. The data presented in Fig 3C was analysed using two way repeated measures ANCOVA. In Fig 5C and 5D data was analysed with an ANOVA using a Dunnett’s post hoc test for comparison between each condition and the cells transfected with pMaxGFP alone. *P < 0.05; **P < 0.01; ***P < 0.001; ns–not significant.
